#  The Volatile Compounds and Bioactivity of *Achillea sieheana* Stapf. (Asteraceae) 

**Published:** 2013

**Authors:** Sevil Albayrak

**Affiliations:** *Department of Biology, Science Faculty, 38039, Erciyes University, Kayseri/TURKEY *

**Keywords:** *Achillea sieheana*, Essential oil, Antimicrobial activity, Antioxidant activity, DPPH

## Abstract

The *in vitro *antioxidant and antimicrobial activities of the essential oil and methanolic extract of *Achillea sieheana *Staf. (Asteraceae) were investigated in this study. The chemical composition of the essential oil isolated by hydro-distillation from the aerial parts of *A. sieheana *was analyzed by GC–MS. Camphor (43.36%), Artemisia ketone (25.95%), 1.8-cineole (6.29%) and camphene (4.77%) were the main components in the essential oil. Their antioxidant activities were also evaluated using phosphomolybdenum, *β*-carotene bleaching and 2,2-diphenyl-1- picrylhydrazyl (DPPH) radical scavenging assays. *A. sieheana *methanolic extract showed an effective DPPH scavenging activity (IC_50 _= 87.04 μg/mL). *The *extract had also a high reducing effect (71.08%) on the oxidation of *β*-carotene. In addition to evaluating the antioxidant activity of this plant, the total phenolic and flavonoid contents were measured in the extract. The antimicrobial activities of the methanolic extract and the oil were also tested against 13 bacteria and two yeasts. The results showed that both had strong antimicrobial activity against the tested microorganisms.

## Introduction

The Reactive Oxygen Species (ROS) are generated as byproducts of biological reactions or from exogenous factors. The ROS induce oxidative damage to various biomolecules including proteins, lipids, lipoproteins and DNA and also play an important causative role in initiation of some diseases such as cardiovascular diseases, rheumatism, diabetes mellitus and cancer (1). Synthetic antioxidants such as butylated hydroxyanisole (BHA), butylated hydroxytoluene (BHT), and tert-butylhydroquinone (TBHQ) have been widely used in food industry to prevent oxidative deterioration, but they are suspected to be responsible for liver damage and carcinogenesis (2). For this reason, there is a growing interest in finding natural antioxidants from plant materials to replace the synthetic ones. Many plant species have been studied in search of novel antioxidants, but there is still a demand to find more information concerning the antioxidant potential of plant species (3). 

Phytochemicals are widely distributed in the plant kingdom and are considered to be biologically active secondary metabolites. These phytochemicals have been shown to be associated with many health promoting effects, such as protection against inflammation, cardiovascular diseases, diabetes, asthma, and cancer (4). Phytochemicals such as phenolic compounds exhibit antioxidant properties due to their high redox potential. They also exhibit a wide range of biological activities including antimicrobial activity, anticarcinogenicity and antiproliferation, and many other biological activities which can be attributed to their antioxidant properties (1).

The genus *Achillea *L. (Asteraceae) is represented by about 85 species mostly found in Europe and Asia and a handful in North America (5). It is represented by 42 species in the flora of Turkey and 23 of them are endemics (6, 7). The species of *Achillea *genus are known in Anatolia as “Civanperçemi”, “Pireotu” and “Yılan çiçeği”. It is used in folk medicine as an appetizer, wound healer, diuretic, carminative or menstrual regulator (8). Also, the aerial parts of different species of the genus *Achillea *L., are widely used in folk medicine due to numerous pharmacologic properties such as anti-inflammatory, antioxidant, antispasmodic, stomachic and antiseptic effects (9, 10).

In the present study, the aerial parts of the endemic *A. sieheana *Stapf used in traditional Turkish medicine were investigated for their essential oil compositions, and the *in-vitro *antioxidant and antimicrobial activities.

## Experimental

*Chemicals*

Folin-Ciocalteu reagent, DPPH, sodium carbonate, Gallic acid, Ascorbic acid, Nutrient agar, Nutrient broth, Malt extract agar and Malt extract broth were purchased from Merck. The other chemicals and solvents used in this experiment were of analytical grade and purchased from Merck. 

*Plant material*

- *sieheana *Stapf was collected from the Develi, Sindelhoyuk, Kayseri, Inner Anatolia region of Turkey in July 2009 (latitude: 38^o^ 21.207 N 35^o^ 20.054 E, and altitude: 1078 m). Plants were collected during their flowering season. They were identified by a senior taxonomist Prof. Dr. Ahmet AKSOY from Erciyes University, Department of Biology. The voucher specimens have been deposited at the Herbarium of the Department of Biology, Erciyes University, Kayseri, Turkey (Voucher no.: AAksoy 2350)

*Extraction*

 Dried aerial parts of the plant were crushed in a coffee grinder for 2 min at room temperature. At 15 sec intervals the process was stopped for 15 sec to avoid over-heating of the sample. Powdered plant samples (10 g) were separately extracted using a Soxhlet type extractor with 100 mL methanol. Thereafter, the extracts were filtered through a Whatman No. 1 filter paper and evaporated to dryness under vacuum at 40^°^C with a rotary evaporator (Rotavator, Buchi, Switzerland; *T*< 40 ^°^C). After determining the yield, the prepared extract was stored at 4^°^C until further analyzed. 

*Isolation of essential oil*

Air dried ground aerial parts of the plants were subjected to steam distillation for 3 h using a Clevenger-type apparatus. The obtained essential oil was dried over anhydrous sodium sulphate and after filtration, stored at 4°C until tested and analyzed. The yield was found to be 1.2 % (v/w). 

*Gas chromatography/ MS analysis conditions*

The composition of the volatile constituents of the material was determined by gas chromatography/mass spectrometry (GC/MS)/quadropole detection analysis using a Shimadzu QP 5050 system (Shimadzu, Duisburg, Germany) fitted with an FFAP (polyethylene glycol+2nitroterephthalate) capillary column (50 m × 0*.*32 mm i.d., film thickness 1.2 μm). The detector and injector temperatures were set at 250^◦^C and 240°C respectively. The temperature of the column was held at 120°C for 1 min and then increased at a speed of 2°C min^−1^ up to 220^°^C and held for 20 min. Helium was used as the carrier gas at a flow rate of 10 psi (split 1 : 10). The injection volume of each sample was 1 μL and the ionization energy was set at 70 eV. Qualitative analysis was based on comparison of the retention times and mass spectra (Wiley, Nist and Tutor Libraries). The composition (%) of the essential oil was computed from the GC peak areas without using any correction factors (11).

*Determination of total phenolics*

The total phenolics contents in plant extracts were determined by a colorimetric assay based on procedures described by Singleton and Rossi (12). Briefly, a 40 μL aliquot of plant extracts dissolved in the same solvent was pipetted into a test tube containing 2.4 mL of distilled water. After mixing the contents, 200 μL of the Folin and Ciocalteu’s phenol reagent and 600 μL of a saturated sodium carbonate solution (20% Na_2_CO_3_) were added. The contents were vortexed for 15 sec and then left to stand at room temperature for 2 h. Absorbance measurements were performed at 765 nm using a Shimadzu 1240 spectrophotometer with gallic acid being used for obtaining the standard curve. The evaluation of phenolic compounds was carried out in triplicate. The results were reported as mean values and expressed as mg of gallic acid equivalents (GAE) /g of dry extract.

*Determination of total flavonoids*

Aluminum chloride colorimetric method was used for flavonoids determination (3). 0.5 mL samples of the extract in methanol were separately mixed with 1.5 mL of methanol, 0.1 mL of 10% aluminum chloride, 0.1 mL of 1 M potassium acetate and 2.8 mL of distilled water. The samples were remained at room temperature for 30 min, and the absorbance of the reaction mixture was measured at 415 nm (by Shimadzu UV-Vis 1240, Japan). The results were reported as mean values and expressed as mg of quercetin equivalents (QE) /g of dry extract.

*Determination of antioxidant activity* *Phosphomolybdenum assay*

The antioxidant activities of the plant extracts were determined by the phosphomolybdenum method of Prieto, Pineda, and Aguilar (13): 0.4 mL of the plant extract (1 mg/mL) was mixed with 4 mL of reagent solution (0.6 M sulphuric acid, 28 mM sodium phosphate and 4 mM ammonium molybdate). The tubes were capped and incubated in a water bath at 95^°^C for 90 min. After the samples were cooled to room temperature, the absorbance of the green phosphomolybdenum complex was measured at 695 nm. In the case of the blank, 0.4 mL of the solvent was used in place of sample. The antioxidant activity was determined using a standard calibration curve with ascorbic acid solutions as standard. The mean of three readings was used and the reducing capacity of the extracts was expressed as mg of ascorbic acid equivalents (AAE)/g extract. 

*β-Carotene bleaching assay*

The ability of the extract to inhibit the bleaching of the *β*-carotene–linoleic acid emulsion was determined (14). *β*-carotene (10 mg) was dissolved in 10 mL of chloroform (CHCl_3_). An aliquot (0.2 mL) of this solution was added into a boiling flask containing 20 mg of linoleic acid and 200 mg of Tween 40. The chloroform was removed using a rotary evaporator at 40^°^C for 5 min. Distilled water (50 mL) was slowly added to the residue with vigorous agitation, to form an emulsion. The emulsion (5 mL) was added to a tube containing 0.2 mL of the essential oil (20 mg/mL) or the extract (1 mg/mL) solution. The test emulsion was incubated in a water bath at 50°C for 2 h, after which the absorbance was measured at 470 nm. In the negative control, the essential oil or the extract were substituted with an equal volume of ethanol. BHT (Butylated hydroxytoluene) and BHA (Buthylated hidroxyanisole) were used as the positive control. 

The percent inhibition was calculated by the following equation:

I% = [1- (Abs_0_ sample − Abs_120_ sample) / (Abs_0_ control − Abs_120_ control)] x 100

*DPPH assay*

Hydrogen atoms or electron-donation ability of the plant extract was measured from the bleaching of the purple-colored methanol solution of DPPH (2,2-diphenyl-1-picrylhydrazyl). This spectrophotometric assay uses the stable radical DPPH as a reagent (15). Fifty microliter samples of various concentrations of the plant extract in the same solvent (0.25 - 3 mg/mL) were added to 1 mL of a 0.1 mM solution of DPPH in methanol. After an incubation period of 30 min at room temperature, the absorbance values were read against a blank at 517 nm. IC_50_ (concentration required to scavenge 50% of the DPPH free radicals) values of the plant extracts were determined graphically. The same procedure was repeated with BHT as the positive control. The measurements were performed in triplicate and the results were reported as the mean values.

Radical scavenging activities were expressed as the percent inhibition of the DPPH radical and were calculated by the following equation:

Inhibition% = (*A*_blank_ – *A*_sample_ /*A*_blank_) x 100

where *A*_blank_ is the absorbance of the control reaction (containing all reagents except the test compound) and *A*_sample_ is the absorbance of the test compound.

*Determination of the antimicrobial activity*

The following microroganisms (obtained from the Department of Food Engineering, Erciyes University, Kayseri, Turkey) were used in this study: *Aeromonas hydrophila *ATCC 7965, *Bacillus brevis *FMC 3, *B. cereus *RSKK 863, *B. subtilis *ATCC 6633, *Escherichia coli *ATCC 25922, *Klebsiella pneumoniae *FMC 5, *Listeria monocytogenes *1/2B, *Morganella morganii*, *Proteus mirabilis *BC 3624, *Pseudomonas aeruginosa *ATCC 27853, *Salmonella typhimurium *NRRLE 4463, *Staphylococcus aureus *ATCC 29213, *Yersinia enterocolitica *ATCC 1501, *Candida albicans *ATCC 1223 and *Saccharomyces cerevisiae *BC 5461.

The antimicrobial activity assay of the extract was carried out by agar-well diffusion method (11). Each microorganism was suspended in sterile nutrient broth. Test yeasts (*C. albicans*, *S. cerevisiae*) were suspended in malt extract broth. Suspensions of the microorganisms, adjusted to 10^6^-10^7^ colony-forming units (cfu)/mL, were placed in flasks containing 25 mL of sterile nutrient or malt extract agar at 45°C. The mix was poured into Petri plates (9 cm in diameter). Then the agars were allowed to solidify at 4°C for 1 h. The wells (5 mm in diameter) were cut from the agar. The extracts were prepared at 1%, 2.5%, 5% and 10% concentrations in the absolute methanol. 50 μL of the extract solutions were applied to the wells. Absolute methanol without extract was used as a control. The antimicrobial activity tests of the essential oils were then carried out by the disc diffusion method (16). 250 μL of suspensions containing 10^6^-10^7^ cfu/mL of microorganisms were placed in flasks containing sterile nutrient or malt extract agar at 45°C. The mix was poured into Petri plates (9 cm in diameter) and the agars were allowed to solidify at 4°C for 1 h. The discs (6 mm in diameter) were impregnated with 10 μL of the essential oil placed on the inoculated agar. *Y. enterocolitica,* *C. albicans *and *S. cerevisiae *were incubated at 25°C for 24-48 h in inverted position. The other microorganisms were incubated at 37°C for 18-24 h. At the end of the incubation period, all plates were examined for any zone of growth inhibition and the diameters of such zones were measured in millimeters. Ampicillin (AMP-10 μg/disc), Chloramphenicol (C-30 μg/disc), Erythromycin (E-15 μg/disc), Gentamicin (CN-10 μg/disc) and Oxacillin (OX-1 μg/disc) (Oxoid) standard antibiotics were used as positive controls. All the tests were performed in duplicate and the results were presented as mean values.

## Results and Discussion

The chemical composition of the hydrodistillated essential oil isolated from the aerial parts of *A. sieheana *was analyzed by GC–MS (Table 1). GC-MS analysis of the essential oil resulted in identification of 22 compounds constituting 96.2% of the total oil. Camphor (43.36%), Artemisia ketone (25.95%), 1.8-cineole (6.29%) and camphene (4.77%) were found to be the predominant components comprising 80.37% of the oil. 

To the best of our knowledge, there are many reports on chemical composition of the oils isolated from plants belonging to the genus *Achillea*. In these reports, cineole, camphor and borneol have been found as major compounds in many other *Achillea *species (5, 17-19). Our results show a strong similarity with these reports. However, in the present study the borneol amount was found to be low in *A. sieheana *oil. Piperitone, camphor and cineole have recently been found to be the major components in the *A. biebersteinii *essential oil (20-23). But, piperitone could not be found in *A. sieheana *oil. 

The chemical composition of *A. sieheana *oil has previously been reported by Tabanca *et al*. (24). In a previous study on the essential oil composition of *A. sieheana*, camphor was determined as the major component (39.9%) along with 108 compounds representing 95.3% of the total essential oil. 1,8-cineole (15.5%) and camphene (8.3%) were also reported as the other major constituents. However, yomogi alcohol, *δ*-elemene, 3.6-dimethyl 2.3.3a.4.5.7a-hexa hydro furan, artemisia alcohol, 2(10)-pinene-3- one, terpienole-4, bicyclo[3.1.1] hept-2-ene-2-carboxaldehyde 6.6-dimethyl, germacrene –D and germacrene –B have not been reported in their study. These differences could be attributed to different geographic regions, climate, altitude, and collection time.

**Table 1:** Composition of A. sieheana essential oil

Compound	RT^b^	%
*α*-pinene ^a^	9.4	1.28 ^c^
Camphene	11.3	4.77
*β*-pinene	13.3	1.03
Sabinene	13.8	0.2
*α*-terpinene	17.1	0.18
Limonene	18.2	0.37
1.8-cineole	18.8	6.29
*γ*-terpinene	21.0	0.46
Artemisia ketone	27.5	25.95
Yomogi alcohol	30.0	2.09
*δ*-elemene	35.6	0.94
3.6-dimethyl 2.3.3a.4.5.7a-hexa hydro furan	36.0	0.23
Artemisia alcohol	37.4	2.47
Camphor	39.5	43.36
2(10)-pinene-3-one	42.1	0.51
Terpienole-4	44.3	1.36
Bicyclo[3.1.1] hept-2-ene-2-carboxaldehyde 6.6-dimethyl	46.0	0.25
trans-pinecarveol	47.7	0.34
*α*-terpineol	50.0	0.39
Borneol	50.5	1.72
Germacrene -D	51.4	1.26
Germacrene-B	53.0	0.75
Total		96.2
	

The amount of total phenolics in the *A. sieheana *methanolic extract was determined according to the Folin–Ciocalteu method and expressed as gallic acid equivalents (Table 2). The mean amount of total phenolics was 15.69 ± 1.2 mg GAE/g of extract. The principle of the aluminum chloride colorimetric method is that aluminum chloride forms acid stable complexes with the C-4 keto group and either the C-3 or C-5 hydroxyl group of flavones and flavonols. In addition, aluminum chloride forms acid labile complexes with the ortho-dihydroxyl groups in the A- or B-ring of flavonoids (25). Total flavonoid content in the *A. sieheana *methanolic extract was expressed as quercetin equivalent (Table 2). The mean amount of total flavonoids was 11.28 ± 0.1 mg QE/g extract.

**Table 2:** The yields, total phenolic content, total flavonoid content, total antioxidant activities, IC_50_ values and the effects on *β*-carotene bleaching of A. sieheana.

	Methanol Extract	Essential oil
Yield (%)	5.77	1.2
Total phenolic content (mg GAE/g extract)	15.69 ± 1.2*	-
Total flavonoid content (mg QE/g extract)	11.28 ± 0.1	
Total antioxidant activity (mg AAE/g extract)	131.73 ± 0.6	-
*β*-carotene bleaching (I%)	71.08 ± 1.9	75.20 ± 2.5
IC_50 _(μg/mL)	87.04	-

*Values expressed are mean ± standard deviation of three experiments. Total phenolic content expressed as gallic acid equivalent (GAE), total flavonoid content expressed as quercetin equivalent (QE), total antioxidant activity expressed as ascorbic acid equivalent (AAE).

-: not determined

This result was different from that of Ardestani and Yazdanparast (26) who reported that total phenolic and flavonoid contents were 104.66 ± 4.39 mg GAE/g and 49.04 ± 1.98 mg catechin equivalent/g in *A. santolina *hydro-alcoholic extract. Total phenolic content of A. *biebersteinii *extract was found to be 51 μg/mg GAE by Baris *et al. *(22). As far as we know the total phenolic and flavonoid contents in the *A. sieheana *methanolic extract are reported here for the first time.

The antioxidative activity of *A. sieheana *was determined by the following three complementary assays: phosphomolybdenum, *β*-carotene bleaching, and DPPH radical scavenging assays. The total antioxidant activity of methanolic extract was determined by using phosphomolybdenum method, which is based on reduction of Mo (VI) to Mo (V) by the antioxidant compound. The total antioxidant activity of the extract was found to be 131.73 ± 0.6 mg AAE/g of dry extract (Table 2). 

In *β*-carotene bleaching assay, the oxidation of linoleic acid generates peroxyl free radicals due to the abstraction of hydrogen atom from diallylic methylene groups of linoleic acid. The free radical then attacks the highly unsaturated *β*-carotene. As a result, *β*-carotene is oxidized and breaks down in part, subsequently losing its chromophore and characteristic orange color, which is monitored spectrophotometrically. Hydroperoxides formed in this system are neutralized by antioxidants from the extracts. Thus, the degradation rate of *β*- carotene depends on antioxidant activity of the extracts (14). The antioxidant activities of the essential oils and extracts were assayed in *β*-carotene–linoleate system and compared with that of BHT and BHA (Table 2). Both the oil and the extract prevented the bleaching of *β*-carotene. The inhibition values of methanolic extract (1 mg/mL), essential oil (20 mg/mL), BHT (1 mg/mL) and BHA (1 mg/mL) were found to be 71.08%, 75.20%, 84.26% and 94.33% respectively. The *β*-carotene bleaching effects of both the extract and the oil were similar to BHT and BHA at tested concentrations.

DPPH has been widely used to evaluate the free radical scavenging activities of various antioxidant substances. DPPH is a stable free radical and accepts an electron or hydrogen resulting in non-radical form. The effects of antioxidants in DPPH radical scavenging test reflect the hydrogen donating capacity of a compound. DPPH radical scavenging activity was expressed as the percent inhibition of initial DPPH absorption by the extract. A decrease in concentration of DPPH radical was observed due to scavenging ability of the methanolic extract and BHT. The extract showed good free radical scavenging capacity at all concentrations studied. The scavenging activity increased with increasing concentration of the extract. The percent inhibitions of the extracts were 3.24%, 9.71%, 17.18% and 33.33% at 8.3, 16.6, 33.3 and 66.6 μg/mL concentrations respectively. The free radical scavenging activity of the extract was significantly lower than that of BHT (92.15% at 66.6 μg/mL). At tested concentration, the percent inhibition of essential oil was not determined because of its low activity. The IC_50 _value of the extract was reported as μg/mL and is shown in Table 2. A lower IC_50_ value indicates a greater antioxidant activity. The IC_50_ value of the extract was found to be 87.04 μg/mL.

There is no previous literature on antioxidant activities of *A. sieheana *extract. However, several studies have been conducted for antioxidant activities of extracts from different *Achillea *species (17, 21, 27). Ardestani and Yazdanparast (26) reported that hydro-alcoholic extract of *A. santolina *had concentration dependent anti-radical activity in DPPH assay and its IC_50 _value was 55 μg/mL which is lower than that of *A. sieheana *methanolic extract. The difference between results could be attributed to the fact that different extraction techniques and plant species were used in these studies. In the present study, the antioxidant activity of methanolic extract was partly similar to that determined by Baris *et al*. (22). In their study, the *A. biebersteinii *methanolic extract showed better free radical scavenging activity (IC_50_ = 89.90 μg/mL) than the essential oil (IC_50_ = 8900 μg/mL). However, Baris *et al. *(22) reported that they were not effectively able to inhibit the linoleic acid oxidation (22.7% and 16% for the extract and the essential oil, respectively) at 2 mg/mL, in the *β*-carotene/linoleic acid assay. Similarly, Maggi *et al*. (28) reported that *A. ligustica *essential oils had a moderate activity in DPPH and *β*-carotene bleaching test. In contrast to our results, Candan *et al. *(5) reported that *A. millefolium *subsp. *millefolium *oil strongly reduced the DPPH radical (IC_50_ = 1*.*56 μg/mL). The antioxidative effect of *A. sieheana *could be due to the flavonoid and phenolic contents. Similarly, it has previously been reported that the infusions prepared form *Achillea *species had an antioxidant capacity, which is consistent with their total flavonoid and phenolic contents (29).

The results obtained in the antimicrobial activity assays of the methanolic extract and essential oil of *A. sieheana *against 13 bacteria and 2 yeasts are shown in Table 3. Absolute methanol (control) had no inhibitory effects on the fifteen microorganisms tested. The antimicrobial activities of both the extract and essential oil are compared with standard antibiotics including Ampicillin (AMP-10 μg/disc), Chloramphenicol (C-30 μg/disc), Erythromycin (E-15 μg/disc), Gentamicin (CN-10 μg/disc) and Oxacillin (OX-1 μg/disc). The extract was shown to possess a broad-spectrum antibacterial activity at 1, 2.5, 5 and 10% concentrations. The extract showed antibacterial activity against the Gram (+) bacteria tested except *B. subtilis*. In addition, the extract showed antibacterial activity against the Gram (-) bacteria tested except *E. coli*, *M. morganii*, *P. mirabilis *and *S. typhimurium*. The most sensitive microorganisms to the *A. sieheana *extract were *B. cereus *and *L. monocytogenes*. No activity against the tested yeasts (*C. albicans *and *S. cerevisiae*) was observed for the extract. The essential oil of *A. sieheana *had great potential for antimicrobial activity against all tested microorganisms except *C. albicans *(Table 3). The inhibition zones were in the range of 7.5-14 mm. The essential oil showed antimicrobial activity against *E. coli*, *M. morganii*, *P. mirabilis*, *S. typhimurium*, *B. subtilis *and *S. cerevisiae *while the methanolic extract was not active against these strains. 

**Table 3:** Antimicrobial activities of methanol extract and essential oil of A. sieheana (inhibition zones. mm).

**Microorganisms**	**Methanol extract (%)**					**Essential**	**Antibiotics (μg)**				
	**10**		**5**	**2.5**	**1**	**oil**	**AMP**	**C**	**CN**	**E**	**OX**
Gram (-)											
*A. hydrophila*	9.0 ± 0.0 ^*^		8.0 ± 0.0	7.5 ± 0.7	7.0 ± 0.0	9.0 ± 0.0 ^a^	27.0 ± 0.0 ^a^	18.0 ± 0.0	8.5 ± 0.7	20.0 ± 0.0	15.0 ± 0.0
*E. coli*	-		-	-	-	8.0 ± 0.0	6.5 ± 0.7	17.0 ± 0.0	9.0 ± 0.0	-	-
*M. morganii*	-		-	-	-	10.0 ± 0.0	-	11.0 ± 0.0	-	-	-
*K. pneumoniae*	8.5 ± 0.7		-	-	-	7.5 ± 0.7	14.0 ± 0.0	13.0 ± 0.0	6.5 ± 0.7	11.0 ± 0.0	-
*P. mirabilis*	-		-	-	-	8.5 ± 0.7	26.0 ± 0.0	19.0 ± 0.0	8.0 ± 0.0	-	-
*P. aeruginosa*	9.0 ± 0.0		8.5 ± 0.7	7.5 ± 0.7	7.0 ± 0.0	8.0 ± 0.0	25.0 ± 0.0	15.0 ± 0.0	12.0 ± 0.0	-	-
*S. typhimurium*	-		-	-	-	9.0 ± 0.0	24.0 ± 0.0	22.0 ± 0.0	8.0 ± 0.0	-	-
*Y. enterocolitica*	7.0 ± 0.0		-	-	-	8.0 ± 0.0	8.0 ± 0.0	17.0 ± 0.0	9.0 ± 0.0	7.0 ± 0.0	-
Gram (+)											
*B. brevis*	10.0 ± 0.0		8.5 ± 0.7	6.5 ± 0.7	-	10.5 ± 0.7	8.0 ± 0.0	20.0 ± 0.0	16.0 ± 0.0	22.0 ± 0.0	-
*B. cereus*	13.0 ± 1.4		10.0 ± 0.0	8.0 ± 0.0	7.0 ± 0.0	10.0 ± 0.0	31.0 ± 0.0	21.0 ± 0.0	11.0 ± 0.0	18.0 ± 0.0	20.0 ± 0.0
*B. subtilis*	-		-	-	-	9.0 ± 0.0	24.0 ± 0.0	25.0 ± 0.0	12.0 ± 0.0	20.0 ± 0.0	19.0 ± 0.0
*L. monocytogenes*	13.0 ± 0.0		10.0 ± 0.0	-	-	9.0 ± 0.0	28.0 ± 0.0	25.0 ± 0.0	13.0 ± 0.0	23.0 ± 0.0	-
*S. aureus*	8.0 ± 0.0	7.0 ± 0.0		-	-	8.0 ± 0.0	16.0 ± 0.0	15.0 ± 0.0	7.0 ± 0.0	12.0 ± 0.0	-
Yeasts											
*C. albicans*	-	-		-	-	-	-	-	-	-	-
*S. cerevisiae*	-	-		-	-	14.0 ± 0.0	-	-	-	-	-

*: Inhibition zones include diameter of hole (5 mm). Sample amount is 50 μL.

a: inhibition zones include diameter of disc (6 mm).

Values expressed are mean ± standard deviation of two experiments.

Ampicillin (AMP-10 μg/disc), Chloramphenicol (C-30 μg/disc), Erythromycin (E-15 μg/disc), Gentamicin (CN-10 μg/disc), Oxacillin (OX-1 μg/disc).

-:Not active

As far as we could ascertain, the antimicrobial activities of the extract and essential oil of *A. sieheana *are reported for the first time. However, similar results for antimicrobial assays of the extract and essential oil of various *Achillea *species have been reported previously. The antimicrobial activities of the essential oils isolated from *A. setacea, A. teretifolia *(19), *A. millefolium *subsp. *millefolium *(5), and *A. teretifolia*, *A. nobilis *subsp. *neilreichii *(17) have previously been reported. Baris *et al. *(22) showed that *A. biebersteinii *essential oil exhibited antimicrobial activity, whereas the methanolic extract was inactive. It has been reported that both the water-insoluble sub-fraction of methanolic extract and the essential oil *of A. sintenisii *were active against some test microorganisms studied (18). Similar results for *A. biebersteinii *were found by the same researchers (21). The antimicrobial activities of the extracts of *Achillea clavennae*, *Achillea holosericea*, *Achillea lingulata *and *Achillea millefolium *against *S. aureus*, *E. coli*, *K. pneumoniae*, *P. aeruginosa*, *Salmonella enteritidis*, *Aspergillus niger *and *C. albicans have been shown by *Stojanović *et al*. (30).

## Conclusion

The results from various antioxidant activity test systems reveal that the methanolic extract and essential oil of *A. sieheana *have significant antioxidant activity. The present study also showed that the methanolic extract of *A. sieheana *had high phenolic and flavonoid contents. Both the extract and essential oil possessed strong antimicrobial activity. They could be useful as potential sources of natural antimicrobial and antioxidant agents in the pharmaceutical and food industries. Results reported here can be considered as the first detailed document on the *in-vitro *antioxidant and antimicrobial activities of *A. sieheana*. However, further investigations should be made for the isolation and identification of individual phenolic compounds. *In*-*vivo *studies are also needed for better understanding of the mechanism of action as an antioxidant.
